# The Role of the Hedgehog Pathway in Chemoresistance of Gastrointestinal Cancers

**DOI:** 10.3390/cells10082030

**Published:** 2021-08-09

**Authors:** Yabing Liang, Ling Yang, Jingwu Xie

**Affiliations:** 1 Inner Mongolia Key Laboratory of Medical Cell Biology, Clinical Medical Research Center of the Affiliated Hospital, Inner Mongolia Medical University, Hohhot 010050, China; 15149009099@163.com; 2 Wells Center for Pediatric Research, Department of Pediatrics, Indiana University School of Medicine, Indianapolis, IN 46202, USA

**Keywords:** the hedgehog pathway, chemotherapy, resistance, gastric cancer, colorectal cancer, pancreatic cancer

## Abstract

The hedgehog pathway, which plays a significant role in embryonic development and stem cell regulation, is activated in gastrointestinal cancers. Chemotherapy is widely used in cancer treatment. However, chemoresistance becomes a substantial obstacle in cancer therapy. This review focuses on the recent advances in the hedgehog pathway’s roles in drug resistance of gastrointestinal cancers and the novel drugs and strategies targeting hedgehog signaling.

## 1. Introduction

The hedgehog (HH) pathway plays a crucial role in embryonic development, tissue homeostasis, and carcinogenesis [1,2]. HH ligands activate signaling by binding to receptor patched 1 homolog (PTCH1). In the absence of HH ligands, PTCH1 prevents smoothened (SMO) from transducing a signal to the downstream glioma-associated oncogene homolog (GLI) transcription factors. HH ligands bind to PTCH1, and relieve PTCH1’s inhibition on SMO, allowing SMO to signal downstream effectors GLI, which activates the target genes via specific genomic DNA sequences (TGGGTGGTC) [3,4].

Activation of GLI proteins via the HH–PTCH1–SMO axis is regarded as the canonical HH signaling pathway. In addition to the canonical pathway, some molecules can bypass the ligand-receptor signaling axis to activate GLI, and these types of regulation are regarded as non-canonical HH signaling. Non-canonical HH signaling is found in malignant diseases. KRAS signaling [5,6], transforming growth factor β (TGFβ) [7], AKT [8], protein kinase C (PKC) [9], and SOX2-bromodomain-containing protein 4(BRD4) [10] are reported to regulate HH signaling via non-canonical pathways.

Chemotherapy is widely used in cancer treatment, and significant improvement is achieved in the prognosis of patients. However, not all patients benefit from it. Chemoresistance becomes a substantial obstacle in cancer therapy due to intrinsic resistance, which occurs at the beginning or even before the treatment, or acquired resistance after initial response to treatment, resulting in relapse [11,12]. Platinum, 5-Fluorouracil (5-FU), and gemcitabine are the most commonly used drugs in the chemotherapy of gastric, colorectal, and pancreatic cancers, and the underlined mechanisms of drug resistance have been studied. Mechanisms of chemoresistance include cancer stem cells(CSCs), tumor microenvironment, and ATP-binding cassette (ABC) transporter family proteins [13,14,15].

Our group studied drug resistance in gastrointestinal cancers and found the HH pathway contributes to drug resistance. This review focuses on recent advances that link the HH pathway to drug resistance in gastrointestinal cancers and examines novel drugs and strategies that may overcome HH-mediated drug resistance.

## 2. Cancer Stem Cells

**Figure 1:**
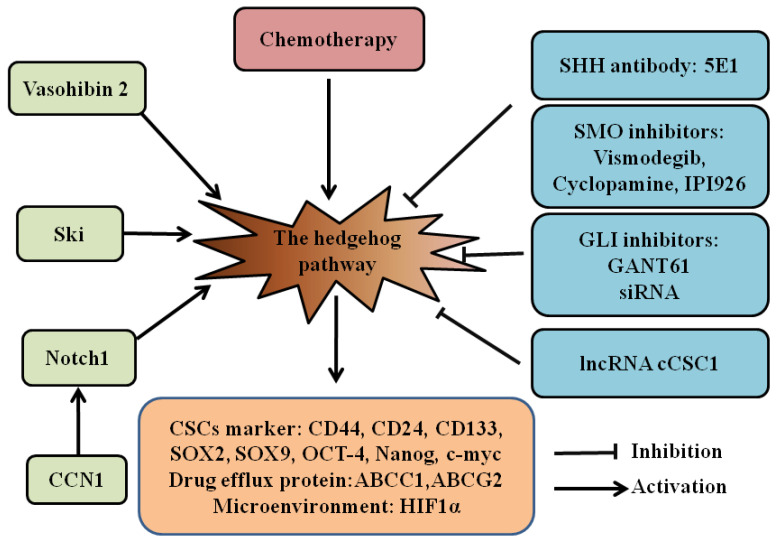
The HH pathway in gastrointestinal CSCs. The role of the hedgehog pathway in drug resistance of gastrointestinal CSCs and inhibitors for hedgehog signaling is summarized. In gastrointestinal CSCs, CCN1, Notch1, Ski, Vasohibin2, and chemotherapy can activate the HH pathway to increase stemness through upregulating CD44, CD24, CD133, SOX2, SOX9, OCT-4, Nanog, and c-myc, to increase drug resistance by elevating the expression of the drug efflux protein ABCC1 and ABCG2, and to adapt to hypoxia via high expression of HIF1α. The HH inhibitors (5E1, vismodegib, cyclopamine, IPI926, GANT61, GLI siRNA, and lncRNA cCSC1) can attenuate these processes. Hedgehog (HH); cancer stem cells (CSCs); cellular communication network factor 1 (CCN1); ATP-binding cassette subfamily C member 1 (ABCC1); ATP-binding cassette subfamily G member 2 (ABCG2); hypoxia inducible factor 1α (HIF1α).

CSCs are a subpopulation of cancer cells capable of self-renewal, metastasis, and treatment resistance. Evidence indicates that CSCs are involved in chemoresistance and relapse of cancers. As a classical developmental pathway, the HH pathway supports the maintenance and survival of CSCs (Figure 1) [16]. Therefore, targeting the HH pathway may be a promising strategy in eradicating CSCs [17,18].

We and others have discovered that the HH pathway is activated in gastric CSCs, which is characterized by a side population [19], cell surface marker CD44 [20,21], CD24, CD133 [22], aldehyde dehydrogenase (ALDH) [23], and Musashi-1 [24]. This pathway is essential for maintaining the viability, motility, and chemotherapeutic resistance of gastric CSCs. Recently, SOX2-positive gastric cancer cells were found to present CSC properties [25]. The CSCs with activated HH signaling contributed to chemotherapy resistance in variant kinds of drugs, such as platinum [19,20], 5-Fu [20,25,26], paclitaxel [27], and doxorubicin [24]. Our group found that the GLI1 protein interacted with the promoter of ATP-binding cassette subfamily G member 2 (ABCG2)through a GLI-binding consensus site in gastric CSCs [19]. ABCG2 is a well-known drug efflux protein and transports small molecules, including chemotherapeutic drugs. This mechanism may explain why gastric CSCs enriched with the HH pathway are more resistant to chemotherapy.

The HH pathway also plays a vital role in colorectal CSCs. Colorectal CSCs showed elevated expression of the genes downstream of HH signaling [28,29], and the HH pathway inhibitor reduced the expression of stemness markers and resistance to 5-FU and platinum in colorectal CSCs [28,30]. Activation of HH signaling is associated with high expression of CD133, SOX9, hypoxia inducible factor 1α (HIF1α), and ATP-binding cassette subfamily C member 1 (ABCC1) in colorectal CSCs [29,30]. lncRNA-cCSC1 inhibited the self-renewal of the colorectal CSCs and reduced their drug resistance to 5-FU by regulating the HH pathway [31]. Culturing three-dimensional organoids becomes a valuable tool to study CSCs by enriching CSCs from cancer cell lines and tissues. Usui et al. established an air–liquid interface (ALI) method to culture organoids from colorectal cancers. These organoids showed resistance to 5-FU and Irinotecan. The HH pathway inhibitors (GANT61) decreased the organoids’ cell viability and inhibited the expression of the CSC markers c-Myc, CD44, and Nanog [32].

Activation of the HH pathway is also found in pancreatic CSCs. Inhibiting HH signaling downregulates Bmi-1 [33], CD133 [34], SOX2 [35], and ABCG2 [33], leading to the reversal of gemcitabine resistance. Hh signaling is also associated with cellular communication network factor 1 (CCN1), Notch1 [36], Vasohibin 2 (VASH2) [37], and Ski [38] in pancreatic cancer. The HH pathway can be activated by VASH2, Ski, and active Notch1 in pancreatic CSCs and contributes to maintain stemness and promote epithelial–mesenchymal transition (EMT) [36,37,38]. Studies also found that combined inhibition of HH and mammalian target of rapamycin (mTOR) signaling effectively reduced pancreatic CSCs [34,39].

Since the HH pathway participates in CSCs, targeting the HH pathway is a promising targeted therapy (Figure 1). In a Phase II trial of advanced gastric cancers, chemotherapy combined with vismodegib was associated with improved survival in patients with high CD44 expression [20]. The sonic hedgehog (SHH) antibody 5E1 reduced the self-renewing capacity of gastric tumorsphere cells and enhanced the efficacy of chemotherapeutic drugs in tumorsphere cells in vitro and in vivo [22]. The SMO inhibitors cyclopamine [22,28,33], IPI-926 [27], and vismodegib [20,24] decreased the stemness of gastrointestinal CSCs in vitro and in vivo. GLI inhibitor GANT61 increased doxorubicin-induced apoptosis in gastric CSCs [24] and regulated drug resistance of colorectal CSCs [29,30]. GANT61 also reduced the sphere formation and cell viability of pancreatic CSCs [34]. Targeting GLI1 using GLI1 siRNA nanoparticles significantly decreased GLI1 protein expression, inhibited gastric CSC tumor spheroid and colony formation, and suppressed cell migration and invasion [40]. Different groups use different types of inhibitors for their studies, from inhibition of HH and SMO to GLI. Moreover, some studies found GLI, not SMO, activated HH signaling in CSCs [34,41]. Therefore, identifying how the HH pathway is activated, caused by HH/SMO, or regulated by other pathways, may help us make sure which component should be targeted and if combined inhibition of the crosstalking pathway is also needed.

## 3. Tumor Microenvironment

**Figure 2:**
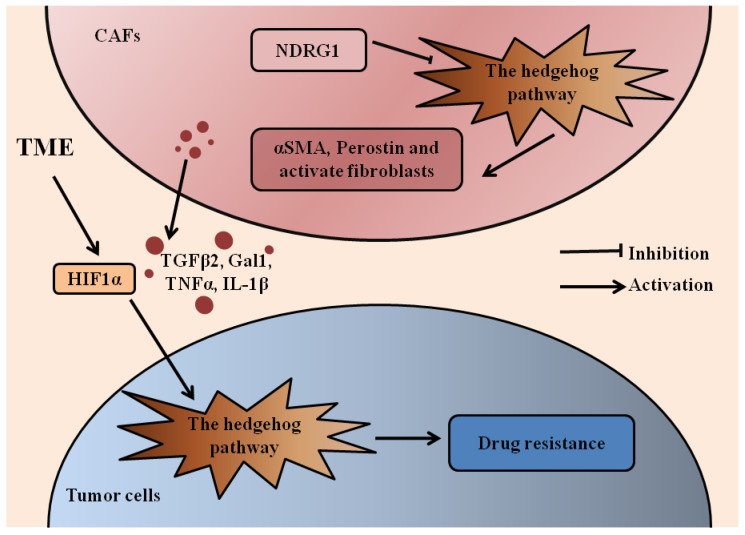
The HH pathway in gastrointestinal cancers and TME. The role of HH signaling in drug resistance of gastrointestinal cancers and CAFs is summarized. Activated HH signaling is found in CAFs with elevated expression of αSMA and periostin. NDRG1 reduces CAF-mediated cell migration and the CAFs’ activation through inhibition of HH signaling. TME-derived HIF1α and CAF-derived TGFβ2, Gal1, TNFα, and IL-1β may activate HH signaling in tumor cells to induce drug resistance. Hedgehog (HH); tumor microenvironment (TME); cancer associated fibroblasts (CAFs); alpha smooth muscle actin (αSMA); N-myc downstream-regulated gene 1 (NDRG1); hypoxia inducible factor 1α (HIF1α); transforming growth factor β2 (TGFβ2); glycan-binding protein galectin-1 (Gal1); tumor necrosis factor α (TNFα); interleukin-1β (IL-1β).

There is mounting evidence to indicate that the tumor microenvironment (TME) is essential for carcinogenesis, angiogenesis, invasiveness, and immune escape [17,42,43]. The TME includes active fibroblasts, immune cells, endothelial cells, neurons, adipocytes, and the extracellular matrix. A hypoxic microenvironment-derived HIF1α and cancer-associated fibroblast (CAF)-derived TGF-β2 activated the expression of the HH transcription factor GLI2 in colorectal CSCs, resulting in increased stemness and resistance to chemotherapy (Figure 2) [44].

Pancreatic ductal adenocarcinoma (PDAC) is characterized by dense stroma that are generally refractory to conventional treatments. Pancreatic stellate cells (PSCs) contribute to this stromal barrier and PDAC progression. The HH pathway plays a vital role in crosstalk between cancer cells and PSCs (Figure 2) [45,46]. Stromal-derived glycan-binding protein galectin-1 (Gal1) [47], tumor necrosis factor α (TNFα), and interleukin-1β (IL-1β) [48] could activate HH signaling in PDAC cells. Moreover, TNFα and IL-1β activated GLI through both SMO and nuclear factor-κB (NF-κB), and SMO inhibition did not altogether abolish GLI activation, indicating that inhibition of canonical and non-canonical HH signaling simultaneously may be more effective in PDAC cells [48]. Activated HH signaling is also found in stromal cells. SHH protein was found co-expressed with markers of mesenchymal cells, alpha smooth muscle actin (αSMA), and periostin [49]. N-myc downstream-regulated gene 1 (NDRG1) reduced PSC-mediated cell migration and PSCs’ activation through inhibition of HH signaling [46]. Our group found that SMO inhibition significantly altered the gene expression profile of the tumor microenvironment but had no significant effects on cancer cell metastasis. The SMO inhibitor combined with the MEK inhibitor showed a reduced number of metastatic nodules in several mouse models for pancreatic cancer [50]. The SMO inhibitor GDC-0449 sensitized PDAC to gemcitabine [45] and doxorubicin [51].

## 4. Crosstalking with Other Pathways

**Figure 3:**
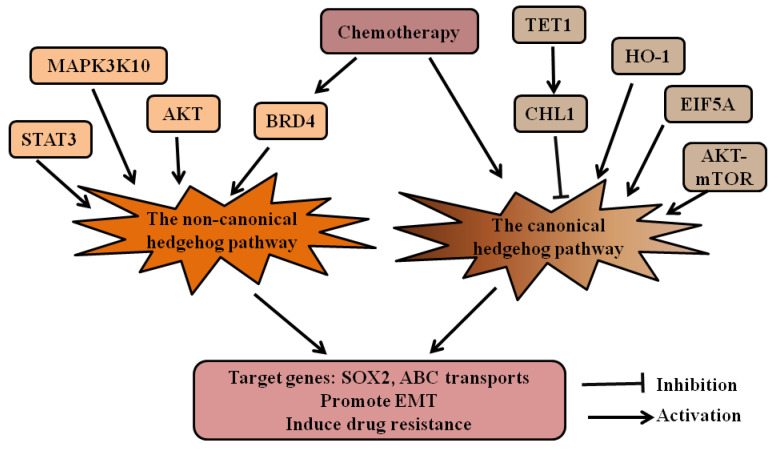
The HH pathway crosstalks with other signaling pathways in gastrointestinal cancers. The crosstalking of the canonical and non-canonical HH pathways with other pathways in drug resistance of gastrointestinal cancers are summarized. In gastrointestinal cancers, HO-1, ATK–mTOR, and EIF5A can activate the canonical HH pathway, while TET1 and CHL1 inhibit the canonical HH pathway. STAT3, MAP3K10, AKT, and BRD4 may activate the non-canonical HH pathway. Chemotherapy activates the HH pathway through different mechanisms. The active HH signaling increases expression of SOX2 and ABC transporters, promotes EMT, and induces drug resistance. Hedgehog (HH); heme oxygenase-1 (HO-1); mammalian target of rapamycin (mTOR); eukaryotic translation initiation factor 5A (EIF5A); signal transducer and activator of transcription 3 (STAT3); mitogen-activated protein kinase 10 (MAP3K10); bromodomain-containing protein 4(BRD4); ATP-binding cassette (ABC) transports; epithelial–mesenchymal transition (EMT).

Studies found that the HH pathway regulates chemotherapeutic resistance by crosstalking with other pathways in many types of cancer, including gastrointestinal cancers (Figure 3). Yao et al. found that GLI1 was activated by the AKT–mTOR pathway in gastric cancer cells. Inhibitors targeting GLI1 and p-AKT may reverse drug resistance and achieve better inhibition than agents targeted against a single molecule [52]. Our group and other researchers found elevated expression of the HH pathway in chemotherapy-resistant colorectal cancer cells [29,53,54,55,56,57]. Besides canonical HH signaling [53,54], GLI was activated by AKT [29] and signal transducer and activator of transcription 3 (STAT3) [55] in colorectal cancers. The activation of the HH signal promoted EMT-related pathways [53,54], and GLI1 could bind to the promoter region of six ABC transporters in colorectal cancers [56].

The most commonly used chemotherapeutic drug for pancreatic cancer is gemcitabine. The role of the HH pathway in gemcitabine resistance is well-studied (Figure 3). Several molecules, including Heme oxygenase-1 (HO-1) [58], mitogen-activated protein kinase 10 (MAP3K10) [59], and eukaryotic translation initiation factor 5A (EIF5A) [60], increased resistance to gemcitabine through activating the HH pathway. TET1 and CHL1 [61] reversed the gemcitabine resistance by downregulating the HH pathway. HH signaling increased resistance to gemcitabine by activating ABCB2 [62]. Our group revealed the GLI–SOX2 signaling axis for regulation of gemcitabine sensitivity and found direct regulation of SOX2 by GLI transcription factors [35]. Gemcitabine treatment also elevated HH signaling, cancer cell stemness, and EMT-related pathways [63] through upregulation of BRD4 [64]. Recent studies found that Dasatinib [65] and Erlotinib [66] resistance was also associated with HH signaling, and inhibition of GLI could reduce the resistance to Erlotinib [66].

## 5. New Drugs and Therapeutic Strategy

The HH inhibitors vismodegib and sonidegib have been approved by the Food and Drug Administration to treat recurrent, locally advanced basal cell carcinoma (BCC) or metastatic BCC, or for those who are not eligible for surgery or radiotherapy. The efficacy and safety of vismodegib and sonidegib have been reviewed in [67]. Vismodegib and sonidegib are also used in clinical trials for other solid tumors (medulloblastoma, prostate cancer, pancreatic cancer, and small cell lung cancer) and hematologic malignancies (actively reviewed in [68,69]).The results from these clinical trials show that the HH inhibitors only promote treatment efficacy in HH-driven cancers.

Since current therapy is still far from satisfactory, novel drugs and new therapeutic strategies were developed to improve the treatment. Novel HH inhibitors also have been developed. GDC0449 analog MDB5 [70] and GLI1 inhibitor NanoHHI [71] overcame SMO mutation and improved the treatment effect. Other drugs, such as curcumin, sensitized colorectal cancer to chemotherapy through downregulating HH signaling [72], and Dpc [46], ormeloxifene [73], Patched 1-interacting peptide [74], and metformin [75] targeted HH signaling was found to reduce the tumor-associated stromal tissue in pancreatic cancers.

Due to dense stromal tissue, chemotherapeutic and targeted drugs, immune cells are hard to get to cancer cells; therefore, targeting stromal cells is a new promising strategy in pancreatic cancers. Since the HH pathway contributes to the development of the dense stromal tissue, several studies combined SMO inhibitors with either cytotoxic chemotherapeutic drugs [76,77,78] or a targeted antibody [79] to increase the delivery of the drugs and promote tumor infiltration of the CD8 T cells. Inhibition of the HH pathway increased intratumoral vasculature density. Some studies found that SMO inhibitors reduced collagen, α-SMA, and GLI-1 expression [76,79]. However, another study found that SMO inhibitor did not decrease the α-SMA-positive fibroblasts and type I collagen in the stroma [77], indicating more studies should be performed to identify the mechanisms how SMO inhibitors increase the delivery of drugs. Furthermore, research suggested that combined the hepatocyte growth factor (HGF)/c-Met and HH pathways inhibitors overcame the resistance to the single-inhibitor treatment and led to sensitization to the gemcitabine treatment [80]. Despite the promising results above and the excellent responses to sonidegib in a mouse model [81], vismodegib does not show improvement in metastatic pancreatic adenocarcinoma (NCT01088815, NCT01064622, [82]). The preclinical model may not accurately reflect the tumor context of patients; patient-derived xenografts, and maybe in the future, patient-derived 3D culture models with tumor cells and a microenvironment, are better materials for studying the efficacy and mechanisms of action of therapeutic drugs.

## 6. Conclusions and Perspectives

Accumulating data suggest that the HH pathway plays an important role in chemoresistance in gastrointestinal cancers. CSCs are the well-known cause for drug resistance and are extensively studied in gastric, colorectal, and pancreatic cancers, and the HH pathway is a promising target for eradicating CSCs. Due to the dense stromal tissue in pancreatic cancers, the role of HH signaling in PSCs is actively being investigated. Inhibition of the HH pathway in PSCs reduces stromal tissue and increases drug delivery, suggesting that HH signaling may also play a mechanical role in chemoresistance. However, studies focused on the HH pathway in the TME of gastric and colorectal cancer chemoresistance are relatively scarce. Despite the different pathological characteristics in gastric, colorectal, and pancreatic cancers, the HH pathway regulates the ABC transporter family proteins in all three types of cancer.

Studies from gastrointestinal cancers and their CSCs provide evidence for the existence of both canonical and non-canonical HH signaling, which do sound the alarm to us. Inhibition of SMO may not inhibit HH activation, and this may partially explain the dismal results of vismodegib in some clinical trials for advanced solid tumors. Identifying how HH signaling is activated, caused by either a ligand-dependent or ligand-independent mechanism, may help us choose the correct inhibitors to attenuate activation of the HH pathway in different cancer contexts.

## Data Availability

Not applicable.
